# Using shanghai municipal data to examine whether student sacrifice exercise in response to academic pressure

**DOI:** 10.1016/j.heliyon.2024.e33527

**Published:** 2024-06-25

**Authors:** Hon Foong Cheah

**Affiliations:** Shanghai University of Sport, School of Economics and Management, China

**Keywords:** Sports expenditures, Adolescent sports activity, Academic pressure, After-school program

## Abstract

The competitive education environment in Mainland China pressure many students to take up after-school lessons, cutting into their leisure time and exercise less, setting a path towards a more sedentary lifestyle. We use a survey on sports consumption for Shanghainese adolescent ages 6 to 17 to examine whether they sacrifice sports activity as they advance through the grade. We found that students a year older exercise 16.2 fewer minutes per week, 1.38 percent less likely to spend on sports training, 1.42 percent more likely to watch sports events, 1.90 percent more likely to buy more sports related goods. Our result shows that students substitute sports activities with more expenditure on sports products and events. When older adolescent exercise less, we are concerned that it may contribute to rising obesity and a more sedentary lifestyle.

## 1 Introduction

Education allows people to secure better careers, improve their living standards, and allow a country's economy to grow and prosper. The Chinese government recognized the importance of an educated society to a growing economy and 1986 mandated that children undergo at least nine years of compulsory schooling [1]. While high school education is not compulsory, good-paying jobs require a degree from a reputable public university, and university admission depends on a student's Gaokao score. This notoriously difficult standardized examination requires students to memorize vast amounts of information. As a result, many parents rely on after-school programs, private tutors, and test preparation programs to better prepare for the tests and to secure their child's future career [2].

When students sacrifice their leisure time to prepare for standardized tests and attend after-school programs, scholars are concerned about the psychological impact on adolescents, such as anxiety and stress. Moreover, when combined with other factors such as university admissions that favor local students, enrollment based on the Chinese household registration system, and the poorly regulated after-school program, Chinese policymakers are concerned about the regional, social, and economic inequality from implementing the Gaokao examination. Between these various factors, the effect of academic pressure on exercise behavior receives very little attention, even though Chinese students have exercised less over the past two decades [3]. Our paper addresses this literature gap by examining the impact on academic pressure and students' exercise propensity. Since exercise behavior is habitual, decisions to exercise less today correlate with future sedentary lifestyles [4]. While our paper does not attempt to draw a connection between academic pressures and adolescents' exercise behavior, we hope that our research topic will draw sufficient attention to future research on this increasingly important topic.

## 2 Literature review

Our research covers three main topics.

1. We discuss economic variables that could affect sports participation.
2. We discuss Chinese adolescents' exercise and sports participation in the past decade.
3. We examine the *Gaokao* examination's adverse negative social, economic, and psychological impact.

### 2.1 Recent research on exercise and sports participation

Most research on exercise primarily focuses on its health benefits. Nevertheless, a growing body of literature has explored economic variables that explain an individual's decision to exercise and engage in sports activities in recent years. Below are several of the variables that relate to our research focus.

***Age and gender on sports participation:*** When it comes to gender, there is a consensus that males participate in sports more than females [5], with males having a higher frequency of participation [6]. For sports participation in different age groups, Ruseski and Maresova [7] found that sports participation decreases with age. In addition, Baumann et al. [8] found that the decline in sports participation with age affects males more than females. García et al. [9] found that sports participation in Spain follows a “U-shaped curve with two peaks: youth and retirement.” These findings support our alternative hypothesis that sports participation increases in age during youth.

***Sports participation and leisure activities:*** The most recent survey by Petilli et al. [10] has shown that students who devote more time to playing video games spend less time on sports. Studies by Kokolakakis et al. [11], Løyland & Ringstad [12], and Downward, P., & Rasciute [13] have found that when facing a time constraint, individuals allocate time between sports and other leisure activities. Therefore, sports and other leisurely activities are substitutes. On the other hand, Adachi and Willoughby [14] found that playing video games leads to greater involvement in sports-related activities, showing that sports and leisure time are complements for certain activities.

*Sports participation and income:* The current literature that explores the effect of income on sports activities indicates mixed results. On the one hand, Downward and Rasciute [15] found that increased household expenditure increases sports participation. On the other hand, Humphreys and Ruseski [16] found no relations or a negative correlation between income and sports participation.

*Peer effects from sports participation:* There is a growing consensus that peer effects partly explain an individual's involvement in sports. Downward and Rascuite [13] show that an individual's choice of physical activities is influenced by the type of sports played by their peers. Downward et al. [17] found that sports participation positively correlates with their parents, implying that parent's involvement is crucial to their offspring's interest in a particular sport.

### 2.2 Exercise and sports participation for Chinese adolescents

Despite the lack of studies that examine the role of academic pressure on exercise, many studies support the benefits of exercise cognitive learning. Many Chinese academics agree that 1.4–2.0 h of physical exercise daily significantly improves adolescents' academic performance, and they should maintain such levels even when faced with academic pressure [18]. A study conducted by Fang and Huang [19] has shown that teenagers who exercise regularly received 0.149 higher cognitive ability scores (in terms of standard deviation) than those who do not exercise regularly. A recent study by Liu et al. [20] has shown that physical exercise positively affects adolescents' mental health.

Despite the benefits of exercise, recent studies show that Chinese adolescents exercise less, causing suboptimal physical and mental health [21]. The most recent report indicates that one-fourth of urban boys are overweight, and the average runtime for boys for 1000 m has dropped by about 15 s compared to the previous decade, with a similar decline in the performance of other track-and-field sports such as long jumps, pull-ups, and sit-ups [3]. Lower sports activities are linked to declining physical health, with a rise in myopia (short-sightedness) rate to 40 percent for elementary students and higher poor blood pressure regulation among Chinese teenagers [21]. As for mental health, Chinese adolescents with mental issues have exceeded the international average range of 15 %–20 %, adversely affecting their relationship with others, ability to control emotions, and learning performance. Less exercise is associated with schoolchildren interacting less with their peers, appearing more self-centered, and less willing to face complex challenges, endure hardships, or cope with setbacks in life [22].

Chinese academics provide several explanations for adolescents' indifference toward sports activities.

1. Individual-level. The pressure from school remains the main factor in explaining the decline in physical activities and sports participation among elementary, middle, and high school students. Other leisure activities such as playing video games, watching television, and watching live broadcasts online can lower participation in physical activities [23].
2. Family-level: The household structure, such as the parents' involvement and attitude towards physical exercise, contributes to their child's physical activities. Studies have found that children exercise less when single parents or grandparents raise them. Parents with at least a college degree are more aware of the importance of physical health and support more youth participation in physical exercise.
3. School-level: The availability of sports facilities in a school compound and the school's emphasis on physical education profoundly impact the schoolchildren's sports activities. Children in schools focusing more on test-oriented education and less on physical education are less likely to exercise. In addition, the availability of education funds and qualified physical education teachers also affects schoolchildren's tendency to exercise [24].
4. Societal-level: Adolescents are less likely to participate in sports when there are few sports venues and public sports facilities within an average walking distance [25].

### 2.3 The adverse socioeconomic and psychological impact from the *Gaokao*

While the current Chinese education system has substantially improved the overall Chinese adult literacy rate, some scholars have criticized certain features of implementing the *Gaokao* examination and university admission policies that create negative socioeconomic impacts. Some of the main criticisms are as follows:

**The proliferation of private tutoring and after-school programs**: Because schools measure student achievement through standardized tests, many parents enroll their children in after-school programs to gain an edge on the test. A survey by Xue and Ding [26] observes that at least 60 percent of urban families are enrolled in after-school tutoring programs. Most after-school programs “cram” students with large amounts of information and undertake many practice tests to get students accustomed to the standardized test. Researchers also found that these programs help students to obtain better scores on their Gaokao examination. Therefore, parents are pressured to enroll their children in an after-school program [27], which increases the cost of education and raising a child, contributing to the demographic problem of an increasingly proportion of aging population [28].

**Psychological pressure:** Despite Kristof's praise for the Chinese education system, he noted that most students complained that the system “kills independent thought and creativity, and envy the American system for self-reliance – and for trying to make learning exciting …” [29]. The exam-oriented education system creates enormous academic pressure to pass the entrance examination, “and those who fail in the entrance examination believe they have few if any career prospects, a belief that de-motivates students” ([30], p. 40). All these create anxiety, clinical depression, and, in rare cases, suicidal tendencies among adolescents. Even during the test, it is common for Gaokao examinees to experience anxiety, with reported cases of fainting in the exam room.

***Regional inequality from unequal university admission:*** Students are discriminated against geographically when a University's admission prefers students from their home region. For example, the prestigious Peking University admits 800 students from a pool of 80,000 students, but only 38 from 660,000 provinces, including Shandong province. Since the best universities in China are located in the wealthiest cities of Beijing, Shanghai, and Guangdong, examinees with fewer advanced educational resources have to compete harder to obtain university admissions. Given the varied admission standards, some families relocate to different cities to advance their children's chances of entering university [31].

## 3 Analytical framework and methods

When analyzing the impact of academic pressure on adolescent's behavior towards sports, we focus on their sports behavior at different years of schooling. The three components of sports behavior are.

1. Frequency of exercise.
2. The propensity to train and watch sports events.
3. Consumption of sports-related goods.

### 3.1 the relation between years of schooling and their frequency to exercise

In economics, a household regards education as an investment in a person's human capital, increasing his ability to contribute to the economy and thus enjoy higher wages [32]. In China, a degree from a prestigious university opens up many job opportunities. Still, university placements require a high score in the college entrance examination, creating a highly competitive academic environment. Students often enroll in after-school and test preparation programs to prepare themselves for the college entrance examination. Moreover, the course material becomes increasingly difficult and requires the help of private tutors. Since private tutoring involves time and money, many students exercise less. Since older students have a greater need to prepare for college entrance examinations, they exercise less than younger students.

On the other hand, the alternative hypothesis is also valid. Theoretically, students who face academic pressure should exercise more to maintain their physical and mental health. Physical activities help students learn better and cope with the immense academic pressure. Since older students face more significant pressure to perform well in the entrance exams, they can exercise more, perhaps at the expense of fewer after-school lessons. Therefore, we propose the following hypothesis and its alternative.

Hypothesis 1 Older students exercise less than younger students.

Hypothesis 1A Older students exercise more than younger students.

### 3.2 the relation between frequency of exercise on sports training and watching sports events

When older students take more private lessons, they have less time for leisure, affecting the time spent exercising, training, and watching sports events. Economic theory does not offer a concrete suggestion. However, when an individual devotes an unchanging proportion to every leisurely activity, less leisure time leads to a uniform decrease in leisure activities, including exercise, training, and watching sports events. On the other hand, the alternative hypothesis is also possible because students may substitute exercise time with more training and watching more sports events. Between these two alternatives, watching more sports events is more plausible because training requires time, which is constrained with less leisure time. Moreover, watching sports events through mobile devices, such as watching match replays and highlights, can be more spontaneous and can be performed at a shorter time interval. In light of these possibilities, we propose two hypothesis and their alternatives below.

Hypothesis 2 Older students spend less on paid sports training.

Hypothesis 2A Older students spend more on paid sports training.

Hypothesis 3 Older students spend less time watching sports events.

Hypothesis 3A Older students spend more time watching sports events.

### 3.3 the relation between exercise and sports-related expenditure

If Hypothesis 1 is correct that older students exercise less because private tutors consume leisure time, we have two possible predictions relating to the consumption of sports-related expenditure. First, less exercise causes students to purchase more sports-related products. Therefore, exercise and sports consumption are substitutes. Alternatively, sports activities complement sports expenditure. Thus, less time on exercise lowers expenditure on sports-related products. We propose the following hypothesis and its alternatives.

Hypothesis 4 Older student make up for lower exercise with more sports-related expenditure (sports expenditure substitutes exercise)

Hypothesis 4A Older student make up for lower exercise with less sports-related expenditure (sports expenditure complements exercise)

### 3.4 the nature of our dataset

Our primary data source originates from a confidential dataset used to construct the Shanghai National Fitness Development Report, also known as “The 300 Index,” which measures the “fitness environment,” “sports participation,” and "physical fitness" of Shanghainese residents. We are motivated to use this dataset because it contains vital information on adolescents' physical activities and spending on sports goods. The data also include basic information on their household income, districts of residence, and their tendency to undergo training and watch certain sports events. It is crucial to control unobserved factors when examining the link between academic pressure and physical activity. While we have received permission to use this dataset for our study, we remove any respondents' personal information from the dataset to remove the possibility of identifying any particular individuals. Since we examine individual's overall behaviors, the lack of personal information has little to no effect on our study.

It is instrumental to examine the intended purpose of this dataset to understand the nature of our dataset. In 2015, the Shanghai Sports Bureau, under the Shanghai Municipal Authority, instructed the School of Economics and Management of the Shanghai University of Sport to conduct an annual survey of its residents to better understand their expenditure on various sports-related activities, from sports equipment to consumption of sports events. The School of Economics and Management forms a committee to draft the questionnaires. After several rounds of discussion, the committee approved the questionnaires, and the School employed a group of interviewers to distribute the questionnaire to selected residential communities and neighborhoods across the Shanghai municipal. Once the interview team collected the survey responses, the dataset was used to construct a 300-Index on and produce the Shanghai National Fitness Development Report available to the public.^1^

When collecting data to compile the Shanghai National Fitness Development Report, interviewers assure each respondent that their personal information will be kept confidential and will not be revealed in the published report. This commitment is crucial to the report's reliability because respondents are likelier to reveal their consumption preferences truthfully when they remain anonymous to the municipal government. Moreover, the National Fitness Development Report is mainly concerned with learning the behavior of the average and median participants. Thus, any individual's information would be of little use in this report.

When the School initiated the survey in 2015, the interviewers encountered several practical issues when collecting individual responses. Therefore, throughout the years, the committee responsible for drafting the questionnaire made minor adjustments to the questionnaire methods to refine the Shanghai National Fitness Development Report. Minor adjustments to the questionnaires may affect comparability between different years.

• In 2016, the School introduced online surveys to all Shanghai residents to widen their coverage. When conducting the online survey, interviewers instruct participants to click on a link that opens a webpage that contains a survey questionnaire. While questions on the website are identical to those on on-site surveys, selection bias may occur because online participants interested in sports are likelier to complete the survey. Nevertheless, online surveys provided a good amount of information since more than half (57 %) of participants were collected from the Internet.
• In 2017, the Shanghai municipal made specific changes to the survey. While the scope of the study remains relatively unchanged, minor differences were made in their questionnaires and the answer choices presented to the respondents. Among these changes include inquiring about the number of hourly exercises per day instead of daily exercises per week and rearranging the list of sports activities in one survey question. Therefore, for specific categories, the data obtained in 2017 cannot be directly comparable to the previous years (2015 and 2016). Nevertheless, as we shall show later, these changes have little to no effect on the respondent's answer, which indicates minimal framing bias.

From 2015 to 2017 (the range of data available for our study), the municipality collected up to 39,000 responses from Shanghai students aged six to seventeen. Table 1 below shows the distribution of the survey respondents, with a large number of online respondents in 2016. While one should be concerned about the non-uniform distribution, we address this issue by performing weighted regression, assuming that individual behaviors are relatively similar within a particular year.

**Table 1:** Distribution of on-site and offline respondents over the three survey years.

Survey type	2015	2016	2017	Total
On-site	6305	4069	6541	16,915
Online	0	18,713	3588	22,301

The questionnaires collected basic information on the district of residence, gender, current schooling grades, sports behaviors such as the amount of daily exercise performed in a week, the type of sports students followed regularly, and their expenditure on sports apparel and footwear, equipment, magazine subscriptions, and tickets to sports events. Table 2, provide a complete description of variables that will be used in the regression analysis.

**Table 2:** Description of the variables used in the regression analysis. For the weighted regressions (SVY), we use survey weights based on the combinatorial clusters of district, year, and online.

Variables	Description
*exercise*	Also referred to as “daily exercise”, this variable record the number of exercise adolescent performed per day in a typical week.
*training*	A dummy variable for adolescents who spend money on renting sports facilities or hiring coaches or sports instructors
*spectator*	A dummy variable for adolescents who spend money on watching sports events
*yos*	An abbreviation of “year of schooling”, this variable represents students' grades, from first to thirteenth, for the 2015 and 2016 surveys,
*school_cat*	In the 2017 survey, instead of a specific grade, this variable represents the student's three schooling categories: elementary, middle, and high school
*apparel and footwear* *expenditure*	A variable that records students' expenditure on sports apparel (jersey, trousers, caps) and footwear for the particular year (in yuan)
*equipment expenditure*	A variable that records students' expenditure on sports equipment for the particular year (in yuan)
*subscription* *expenditure*	A variable that records students' expenditure on sports journals, magazines, and book subscriptions for the particular year (in yuan)
*training expenditure*	A variable that records students' expenditure on the rental of sports facilities and fees for hiring sports instructors (in yuan)
*tickets expenditure*	A variable that records the students' expenditure on tickets and season passes (in yuan)
*other expenditure*	A variable that record the students' other sports related expenditure (including sports tourism) in yuan.
*total expenditure*	The sum of all six expenditure variables: apparel and footwear, equipment, subscription, training, tickets, and other expenditures.
*male*	A dummy variable for male respondents
district*	A variable that indicates the respondent's residence within the sixteen districts in the Shanghai Municipal.
year*	A categorical variable that indicates the year the survey is performed
online*	A dummy variable on whether the study was conducted via the Internet

### 3.5 Regression framework

We perform four regression frameworks to test out the four hypotheses. We use the following framework to analyze the impact of academic pressure on student's propensity to exercise (hypothesis 1).

(1) $$exercise=\beta_{10}+\beta_{11}yos+\Gamma X_{1}+\varepsilon_{1}$$

The variable *exercise* refers to the days of exercise students performed in a week, while *yos* refers to the current year of schooling, from grade 1 (first year elementary) to grade 13 (first year of college). The matrix *X*_*1*_ controls for factors that affect daily exercise level, such as an individual's interest in sports gender, district, and sports-related expenditure. First, our controls include dummy variables on whether an individual pays for sports training or for watching sports events because these factors strongly predicts a person's propensity to exercise. We also include gender into our control because sports participation is typically higher for males, district to control the sporting amenities, income and leisure choices available in a particular district, and sports-related expenditure since exercise relates to an individual's tendency to spend on sports. Finally, the variable *ε*_*1*_ represents unobservable factors that influence a student's decision to exercise.

We use the following framework to examine the impact of academic pressure on students’ propensity to spend on sports training (hypothesis 2),

(2) $$training=\beta_{20}+\beta_{21}yos+\Gamma X_{2}+\varepsilon_{2}$$

*where* training is a dummy variable on whether the respondent spends money on sports programs, while yos refers to the respondent's current years of schooling. Matrix X_2_ contains controls similar to X_1_, except that it includes daily exercise and omits the training variable. Similar to Equation (1), we control for individual's preference towards sports, gender, district, and sports expenditure to partial out the effect of years of schooling on training. Finally, ε_2_ represents unobservable variables influencing an individual's decision to pay for training.

We use the following framework to examine the impact of academic pressure on students’ propensity to follow sports events (hypothesis 3),

(3) $$spectator=\beta_{30}+\beta_{31}yos+\Gamma X_{3}+\varepsilon_{3}$$

where *spectator* is a dummy variable on whether the respondent follows a particular sports event, and *yos* refers to the respondent's current years of schooling. The matrix X_3_ is similar to X_1_ except that it includes daily exercise and omits a dummy variable on spectators. Finally, the variable *ε*_*3*_ represents unobservable variables influencing an individual's decision to follow an event.

The 2017 survey data only indicates if the students belong to elementary, middle, or high school education, as opposed to a specific year of schooling. While we cannot examine yearly changes in a student's grades, we can still investigate the difference in behavior between these schooling categories. Therefore, we modified the initial framework to replace year of schooling with the three schooling categories (elementary, middle, and high school).

(4a) $$exercise=\beta_{10}+\beta_{12}school\_cat+\Gamma X_{1}+\varepsilon_{1}$$

(4b) $$training=\beta_{20}+\beta_{22}school\_cat+\Gamma X_{2}+\varepsilon_{2}$$

(4c) $$spectator=\beta_{30}+\beta_{32}school\_cat+\Gamma X_{3}+\varepsilon_{3}$$

Lastly, we use the following framework to examine the pattern of student's expenditure on sports-related goods when facing academic pressure (hypothesis 4),

(5) $$expenditure=\beta_{40}+\beta_{41}yos+\Gamma X_{4}+\varepsilon_{4}$$

Where expenditure refers to sports-related expenditures and *yos* refers to the respondent's current schooling year. The matrix *X*_*4*_ controls individual preferences for sports, such as daily exercise and propensity to pay for sports training or to watch an event. Other control variables included are gender, and district for similar reasons discussed in Equation (1). Lastly the variable *ε*_*4*_ includes unobservable variables influencing an individual's decision to follow an event.

## 4 Results

### 4.1 Descriptive statistics on sports behavior of Shanghai residence

**Fig. 1:**
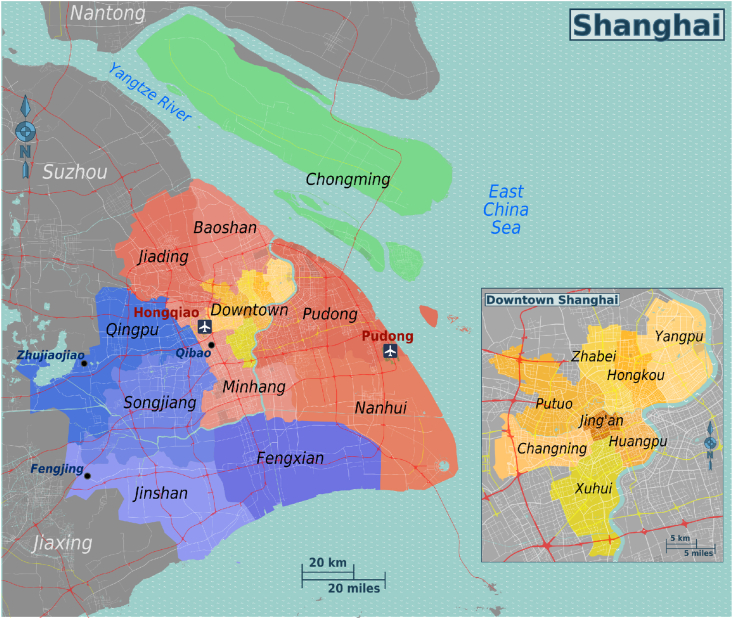
Map of Shanghai Municipal.

Since the offline questionnaires were randomly distributed across the residential area in Shanghai, the composition of urban and rural areas in a district serves as a reliable proxy for household income and the difference in development between rural and urban residents and its effects on sports behavior. Even though the central districts lie between the Yangtze and the Huangpu Rivers, the Shanghai administration area covers a much larger geographical area of almost 2500 square miles, a home to nearly 26 million residents. The sixteen administrative districts are shown in Fig. 1 below. Seven of the sixteen districts are highly urbanized central districts of Huangpu, Xuhui, Changning, Jingan,^2^ Putuo, Hongkou, and Yangpu. As China underwent economic reform during the 1990s, the Shanghai administration developed its eastern frontier of Pudong, a relatively large district encompassing the northern urban areas adjacent to the city center and the south suburban and rural areas of the former Nanhui District. The three outer districts of Minhang, Baoshan, and Jiading benefited immensely from the economic reform and are classified as suburban areas. The remaining five outer districts of Jinshan, Songjiang, Qingpu, Fengxian, and Chongming were located much further away from the city center and had a mix of a suburban and rural landscape.^3^

We divide the districts of Shanghai into four categories.

1. The urban “central” seven inner districts
2. The urban-suburban areas of “Pudong "
3. The "suburban" districts of Minhang, Baoshan, and Jiading
4. The "remote suburban" districts of Jinshan, Songjiang, Qingpu, Fengxian, and Chongming

These four categories represent the four levels of development within Shanghai that proxy the level of affluence and the amenities offered to its residents. Table 3 below depicts the average sports-related expenditure for offline participants according to these four categories. Sports-related expenditures include apparel and footwear, equipment, magazine subscriptions, ticket sales, and travel to sports events. Column 2 shows the average expenditure within each region. Respondents from central districts and Pudong spend more than those residing in the more remote suburban districts. The following three columns, 3 to 5, compute the average expenditure each year of survey data. We find a steady increase in sports expenditure from 2015 to 2017. However, without controlling for the respondents' income levels, we cannot be sure if the expenditure increase is due to the respondents' higher incomes or greater interest in sports-related products.

**Table 3:** Average total expenditure for offline participants.

Region	Average Total Expenditure	2015	2016	2017
Central	3562	3088	4193	3745
Pudong	3950	3242	2986	5820
Suburban	2890	2964	2271	3389
Remote-Suburban	2706	2893	2249	2552

Addressing the concerns of selection bias, Table 4 tabulates the average expenditure for online respondents. Since respondents online are not randomly selected, respondents with a greater interest in sports are more likely to complete the survey. In column 2, the average expenditure for online participants was almost three times higher than their offline counterparts. This result shows that online respondents have a higher income or that only respondents spend more on sports-related products than their offline counterparts. While it is very likely that respondents interested in sports have a higher income, we find a 64 to 78 percent increase in average total expenditure from the four regions from 2016 to 2017, which suggests that those more interested in sports are more likely to complete the survey. While we should be concerned about selection bias from online participants, we include their responses in our analysis because they represent most of the responses. More importantly, *the*y support th*e alternative hypothesis more* because these students are more likely to remain more active in light of academic pressure.

**Table 4:** Average total expenditure for online respondents.

Region	Average Total Expenditure	2016	2017
Central	10441	9023	15518
Pudong	7206	6559	10908
Suburban	11028	6958	11432
Remote- suburban	6606	6166	11027

Nevertheless, we should exercise caution with the average sports expenditure because the residents vary significantly in consumption behavior. At least 13 % of the respondents report zero sports-related expenditure. Even when we account for positive expenditure level, most respondents spend well below the average, which means that a small minority spending far above the average, skewing the average well above its median value. Table 5 tabulates the total expenditure at different percentiles. The median expenditure (50th percentile in column 4) is within 22 %–33 % of the average spending (column 2). The skew in the expenditure distribution is so pronounced that the average expenditure is higher than those in the 75th percentile (column 5). The high average figures are due to the enormous spending on a few residences. As shown in column 6, the expenditure of the 95th percentile is multiple times higher than the average expenditure.

**Table 5:** Total expenditure at different percentiles.

Region	Mean	25th percentile	50th percentile	75th percentile	95th percentile
Central	9520	800	2100	6000	31800
Pudong	5689	780	1700	4868	21600
Suburban	5805	800	1920	4338	17800
Remote- suburban	6224	730	1845	4456	19200

The survey also asks about the exercise choices of students. Table 6 below shows the primary and secondary exercise choices for data from 2015 to 2016, which reported running, walking, and "recreational games" as popular primary exercise choices, followed closely by basketball, badminton, and swimming. The 2017 questionnaire omits the vague choice of "recreational games" and reorders "running and walking" towards the end of the list. Table 7 tabulates the survey results for 2017. These changes may create a potential framing bias in which respondents reply differently because of changes in the questionnaire selection. We find framing bias to be minimal. Even though the 2017 questionnaire removes the ambiguous choice of "recreational games," badminton, basketball, swimming, and soccer were still the preferred modes of exercise, followed closely by "running and walking." Moreover, the primary and secondary exercise choice changes little from 2015 to 2017, showing a stable preference for these sports activities between these three years.

**Table 6:** Popular choices of exercise based on older questionnaires (2015 and 2016).

Mode of exercise	Primary (2015, 2016)	Secondary (2015,2016)
Running	11.62 %	8.71 %
Walking	10.56 %	7.28 %
Recreational games	9.70 %	3.06 %
Basketball	8.05 %	9.06 %
Badminton	7.92 %	11.93 %
Swimming	7.16 %	9.39 %
Soccer	6.28 %	5.79 %
Rope skipping	4.44 %	5.31 %
Table tennis	3.55 %	4.73 %

**Table 7:** Popular choices of exercise based on newer questionnaires (2017).

Mode of exercise	Primary (2017)	Secondary (2017)
Badminton	28.26 %	3.59 %
Basketball	11.15 %	15.22 %
Soccer	9.42 %	9.94 %
Swimming	7.48 %	12.03 %
Running, walking	3.94 %	4.63 %
Table tennis	3.43 %	5.63 %
Rope skipping	3.10 %	5.75 %

Due to inadequate physical activities in public schools, some students enroll in private sports training programs. The survey shows that 41.4 % of the participants pay for private training and instructions. Table 8 below shows the primary choice of paid instruction under the old and new questionnaires. Even though the questionnaires are worded differently, swimming lessons remain the most popular choice (15.4–18.1 %), followed by badminton, basketball, and soccer classes.

**Table 8:** Primary choice of sports training for participants ages 6–17 under old (2015, 2016) and new (2017) questionnaires.

Primary choice for sports training	Old questionnaires (2015,2016)	New questionnaires (2017)
Swimming	18.10 %	15.43 %
Basketball	8.69 %	10.65 %
Soccer	7.09 %	9.55 %
Badminton	6.91 %	14.64 %

The survey also collects information on sports events watched by students. Most students watch sports on their television, mobile devices, on the computer, or at the arena. In addition to the public broadcasts, 31.9 % of participants spend on tickets and subscriptions to follow up on their favorite sports. Table 9 below shows the primary and secondary types of sports watched by Shanghai students aged 6 to 17. Football and basketball were the primary sports events, followed closely by badminton matches, with table tennis, swimming, and tennis events trailing behind.

**Table 9:** The main sports watch by respondents ages 6-17.

Sports watched	Primary	Secondary
Football	18.67 %	4.69 %
Basketball	12.25 %	18.04 %
Badminton	8.11 %	10.17 %
Table tennis	6.34 %	7.16 %
Swimming	6.00 %	8.84 %
Tennis	5.18 %	5.24 %

### 4.2 the effect of academic pressure on student's frequency of exercise

Table 10 displays the regression results from Equation (1), which examines the number of weekly exercises with the independent variables listed in Column 1. Column 2 shows the result using ordinary least squared (OLS) regression without including other control variables. The OLS coefficient of −0.0169 means that, on average, students exercise 0.0169 days fewer than those from the previous grade. While this effect is small since a typical student has three to 5 h of leisure a day, the difference becomes noticeable between two very different grades level. For example, a sixth-grade student spends 1.62 more hours on exercise than a twelfth-grade student. In column 3, we reran the OLS regression after controlling for individual interest in sports by including two dummy variables: 1) whether an individual engages in sports training and 2) a spectator of any sports events. These two variables are strong predictors of a student's decision to participate in physical activities, and the coefficients in lines 1 and 2 of column 3 indicate that, respectively, students who spend on sports training engage in 0.246 more days of exercise and students who spectate sports engage in 0.401 more. Both effects are large and statistically significant. Controlling individual tendencies to engage in sports reinforces the pattern that daily exercise is decreasing in a student's age. For example, a sixth-grade student spends 1.84 additional hours (1.15 waking days) towards exercise compared to a twelfth-grade student.

**Table 10:** Daily exercises at different years of schooling (Standard errors in parentheses.

Dependent variable: Amount of exercise days per week				
	OLS	OLS	SVY	SVY
*yos*	−0.0169∗∗∗ (0.0025)	−0.0192∗∗∗ (0.0025)	−0.0208∗∗∗ (0.0025)	−0.0212∗∗∗ (0.0025)
*training*		0.2457∗∗∗ (0.0198)	0.2461∗∗∗ (0.0198)	0.2392∗∗∗ (0.201)
*spectator*		0.4012∗∗∗ (0.0203)	0.3705∗∗∗ (0.0205)	0.3593∗∗∗ (0.0206)
*male*			0.1760∗∗∗ (0.174)	0.1726∗∗∗ (0.0175)
*Pudong*			−0.1830∗∗∗ (0.0269)	−0.1600∗∗∗ (0.0273)
*suburban*			0.0083 (0.0239)	0.0086 (0.0239)
*remote-suburban*			0.0748∗∗∗ (0.0216)	0.0801∗∗∗ (0.0217)
*apparel and footwear* *expenditure* (‘000 yuan)				0.0061∗ (0.0028)
*equipment* *expenditure* (‘000 yuan)				0.0052∗ (0.0026)

**p < 0.01.

∗ p < 0.05.

∗∗∗ p < 0.001).

In Table 10, column 4, we performed a weighted regression (SVY) based on the survey weights that assume that respondent's behavior clusters around a particular district, the survey year, and whether the survey is conducted online or directly from respondents. The weighted regression includes additional controls such as gender and location [33]. We find that male students, on average, spend an additional 0.177 days more exercise than females (column 4, line 4). We also find that, on average, students in suburban and remote suburban districts exercise more than those in the central districts, possibly because even with higher income and better sports facilities, students in the central districts exercise less because they have more choices for leisure activities. Finally, column 5 reran the weighted regression to include controls on students' sports expenditures such as attire, equipment, magazines, and tickets. The negative impact of academic pressure on exercise remains robust at −0.021 days (column 5, line 1), meaning that a twelfth-grade student, on average, exercises 2.0 h per week more than a sixth-grade student. While we show that students who spend more on sports attire and equipment exercise more, the results are small and weakly significant. The coefficients in column 5, lines 8 and 9 suggest that students who spend an additional 1000 yuan on sports attire and equipment increase their weekly exercise by 0.0061 days and 0.0052 days, respectively. In short, expenditure is a weak predictor of an adolescent's exercise frequency after controlling for other factors.

### 4.3 the effect of academic pressure on the propensity to undertake training

Table 11 displays the regression results from Equation (2), which examines students’ propensity to spend on sports training at different years of schooling. Column 2 shows the result of an OLS regression. Assuming a linear probability framework, the propensity to spend on sports training decreases by 1.38 percent for each year of schooling, which means that, on average, a twelfth-grade student is 8.3 percent less likely to spend on sports training than a sixth-grade student. Column 3 shows the results once we reran the regression controlling for individual-specific interest in sports. We find that each year of schooling reduces the propensity to spend on sports by 1.95 percent for each year of schooling (column 3, line 1), but being a sports spectator increases the chance of spending on sports training by about 43.8 percent (column 3, line 2). Those who exercise an additional day in their weekly routine are 2.2 percent more likely to engage in sports (column 3, line 3). In column 4, even after assuming that individual behaviors are correlated within a particular district, year of survey, and method of collecting survey results, and after controlling for gender and location, we still find that adolescent propensity to spend on training decreases by 1.98 percent for every year of schooling (column 4, line 1). We also find that the tendency to spend on sports training differs with gender, as male students are 1.9 percent more likely to spend on training than female students (column 4, line 4). We also find that respondents living in suburban and rural suburban areas are less likely to spend on sports training because of the fewer availability of sports facilities. Column 5 controls individual spending behavior to find that those who spend 1000 yuan more in training per year will have a 0.65 percent higher chance of spending for training.

### 4.4 the effect of academic pressure on the propensity to watch sports events

Table 12 displays the regression results from Equation (3), which examines respondents’ likelihood of watching the game for students in different years of schooling, using controls similar to those of the previous two analyses. Column 2 shows that the propensity to watch sports events increases by 1.42 percent for every increase in schooling years (column 2, line 1). The effect is large (2 percent, column 3, line 1) once we control for an individual's preference towards sports in column 3, which means that a twelfth-grade student is 12 percent more likely to spend on training than a sixth-grade student. We also find that participating in sports training increases the chance of watching the game by almost 40 percent (column 3, line 1). Column 4 assumes a cluster sample and controls gender and location. Male students are 6.5 percent more likely to watch a sports event (column 4, line 4). Column 5 controls for individual spending behavior and finds that expenditure on tickets and subscriptions to sports tickets weakly correlate with an individual's propensity to spectate sports events. On average, those who spend an additional 1000 yuan on ticket sales have a 0.5 percent higher chance of watching sports events (column 5, line 8).

Table 13 replicates the methods of the previous three regressions (column 5 of Table 10, Table 11, Table 12) for 2017 data using schooling categories instead of specific schooling levels (Equations (4a), (4b), (4c))). We find that in the second row of Table 13, the results match those from the previous data that when students advanced into a higher schooling category (from elementary to middle school), students engaged in fewer daily exercises (column 2) and participated less in sports training (column 3) and watch more sports events (column 4).

**Table 11:** Spending on sports training at different years of schooling. (Standard errors in parentheses.

Dependent variable: Spending on sports training				
	OLS	OLS	SVY	SVY
*yos*	−0.0138∗∗∗ (0.0008)	−0.0195∗∗∗ (0.0008)	−0.0198∗∗∗ (0.0008)	−0.0199∗∗∗ (0.0008)
*spectator*		0.4388∗∗∗ (0.0057)	0.4306∗∗∗ (0.0058)	0.4218∗∗∗ (0.0059)
*exercise*		0.0223∗∗∗ (0.0018)	0.0222∗∗∗ (0.0018)	0.0216∗∗∗ (0.0018)
*male*			0.0193∗∗∗ (0.0053)	0.0167∗∗ (0.0053)
*Pudong*			−0.0275∗∗∗ (0.0083)	−0.0248∗∗ (0.0084)
*suburban*			−0.0801∗∗∗ (0.0072)	−0.0786∗∗∗ (0.0071)
*remote-suburban*			−0.0726∗∗∗ (0.0083)	−0.0674∗∗∗ (0.0084)
*Training expenditure* (‘000 yuan)				0.0064∗∗∗ (0.0009)

*p < 0.05.

∗∗ p < 0.01.

∗∗∗ p < 0.001).

**Table 12:** Spending on sports training at different age groups. (Standard errors in parentheses.

Dependent variable: Sport spectator				
	OLS	OLS	SVY	SVY
*yos*	0.0142∗∗∗ (0.00080)	0.0201∗∗∗ (0.00255)	0.0186∗∗∗ (0.00073)	−0.0183∗∗∗ (0.00073)
*training*		0.3973∗∗∗ (0.0053)	0.3867∗∗∗ (0.0053)	0.3795∗∗∗ (0.0054)
*exercise*		0.0329∗∗∗ (0.0016)	0.0301∗∗∗ (0.00162)	0.0291∗∗∗ (0.00164)
*male*			0.0658∗∗∗ (0.0050)	0.0614∗∗∗ (0.0050)
*Pudong*			−0.1292∗∗∗ (0.0072)	−0.1220∗∗∗ (0.0074)
*suburban*			−0.0489∗∗∗ (0.0067)	−0.0462∗∗∗ (0.0067)
*remote-suburban*			−0.0585∗∗∗ (0.0062)	−0.0591∗∗∗ (0.0062)
*tickets* *expenditure* (‘000 yuan)				0.0051∗∗∗ (0.0007)
*subscription* *expenditure* (‘000 yuan)				0.0033∗∗∗ (0.0008)

*p < 0.05, **p < 0.01.

∗∗∗ p < 0.001).

**Table 13:** Weekly exercise, training and watching sports event at different years of schooling using 2017 data (Standard errors in parentheses.

Dependent variable	Weekly exercises	Sport training	Sport spectator
*School_cat*	−0.2047∗∗∗ (0.0181)	−0.0655∗∗∗ (0.0056)	0.0551∗∗∗ (0.0050)
*exercise*	N/A	0.0169∗∗∗ (0.0031)	0.0153∗∗∗ (0.0028)
*training*	0.1839∗∗ (0.0333)	N/A	0.3360∗∗∗ (0.0090)
*spectators*	0.2056∗∗∗ (0.0371)	0.4170∗∗∗ (00107)	N/A
*male*	0.0740∗ (0.0318)	0.0265∗∗ (0.0095)	0.1285∗∗∗ (0.0082)
*Pudong*	0.0947∗ (0.0420)	−0.0449∗∗∗ (0.0129)	−0.1218∗∗∗ (0.0108)
*suburban*	0.2924∗∗∗ (0.0453)	−0.0173 (0.0140)	−0.0894∗∗∗ (0.0125)
*remote* *suburban*	0.1869∗∗∗ (0.0401)	−0.1124∗∗ (0.0120)	−0.0979∗∗∗ (0.0104)

∗ p < 0.05.

∗∗ p < 0.01.

∗∗∗ p < 0.001).

### 4.5 the effect of academic pressure on sports expenditure

The results from Table 10, Table 11, Table 12, Table 13 indicate that while older students spend less time on exercise (Table 10, line 1; Table 12, column 2, line 1) and on sports training (Table 11, line 1; Table 11, column 3, line 1), they make up for it by watching more sports events (Table 12, line 1; Table 11, column 4, line 1). In other words, since leisure prices rise from higher school workloads, students substitute sports activities with watching more sports events and purchase more sports-related expenditures. However, we should be cautious with such a correlation because the substitution effect may also reflect the individual's changing preference as they age or when parents become less restrictive on their child's expenditure as the child ages. Table 14 below uses data from 2015 to 2016 to examine the role of schooling on the natural logarithm of total spending (Equation (5)). Column 2 employs a simple ordinary least-squared regression, showing that total expenditure increases by about 1.9 percent from a year of schooling. This effect increases to 1.98 percent once we consider the survey design and control for individuals' propensity to engage in sports in column 3. Since individual expenditures cannot take a negative value and nontrivial respondents choose not to spend any on sports-related expenditures, a Tobit specification [34] is more appropriate than linear regression. Using the Tobit specification,^4^ we find that the result remains robust: students a year older on average increase their total expenditure by 1.97 percent. The effect is statistically significant with the inclusion of gender and location (column 5).

**Table 14:** Total sports expenditure at different years of schooling (Standard errors in parentheses.

Dependent variable: Log of Total sports-related expenditure				
	OLS	SVY_OLS	SVY_Tobit	SVY_Tobit
*yos*	0.0190∗∗∗ (0.0030)	0.0198∗∗∗ (0.0029)	0.0197∗∗∗ (00029)	0.0199∗∗∗ (0.0029)
*training*		0.7459∗∗∗ (0.0206)	0.7459∗∗∗ (0.0206)	0.7387∗∗∗ (0.0208)
*spectator*		0.4916∗∗∗ (0.0221)	0.4916∗∗∗ (0.0221)	0.4796∗∗∗ (0.0223)
*exercise*		0.0774∗∗∗ (0.0066)	0.0774∗∗∗ (0.0066)	0.0748∗∗∗ (0.0066)
*male*				0.1420∗∗∗ (0.0188)
*Pudong*				0.0619∗ (0.0279)
*suburban*				−0.0245 (0.0257)
*remote-suburban*				0.0126 (0.0237)

**p < 0.01.

∗ p < 0.05.

∗∗∗ p < 0.001).

The following analysis breaks down the total expenditure into its components: sports apparel and footwear, sports equipment, subscriptions and journals, training, tickets, and other related The following analysis breaks down the total expenditure into its components: sports apparel and footwear, sports equipment, subscriptions and journals, training, tickets, and other related spending, including sports tourism. Table 15 replicates the previous regression framework (column 5 of Table 14). It replaces the dependent variable of total expenditures with six sports-related spendings: sports apparel and footwear (column 2), sports equipment (column 3), sports books, newspapers or magazine subscriptions (column 4), sports training (renting sports facilities and coaches) (column 5), sports events (tickets and season passes) (column 6), and expenditure on travel and other sports-related activities. We observe two notable findings. First, from line 2, as expected, individuals who paid for sports training spend more on all sports-related expenditures. Second, students alter their sports consumption as they age, spending more on sports equipment, subscriptions to magazines and sports events, a small decrease in sports training, and insignificant changes to sports apparel and footwear. While we should be cautious with the insignificant coefficients in these results, this result is still consistent with our findings that older students train less and spend more on watching events, books, and magazine subscriptions. However, the increase in spending is at odds with devoting less time to exercise and sports as they age. Older students may spend more on fewer but better equipment, preferring quality to quantity. However, this is only a speculation since the survey data did not measure the quality of sports equipment.

**Table 15:** Components of sports-related expenditure at different years of schooling. (Standard errors in parentheses.

Dependent variable	*apparel and footwear* *expenditure*	*equipment expenditure*	*subscription expenditure*	*training expenditure*	*tickets expenditure*	*other expenditure*
*yos*	0.0046 (0.0036)	0.0226∗∗∗ (0.0036)	0.0499∗∗ (0.0042)	−0.0085∗ (0.0043)	0.0239∗∗∗ (0.0043)	0.0012 (0.0048)
*training*	0.2938∗∗∗ (0.0265)	0.4552∗∗∗ (0.0276)	0.4766∗∗∗ (0.0331)	0.8751∗∗∗ (0.0335)	0.5040∗∗∗ (0.0346)	0.5722∗∗∗ (0.0387)
*spectator*	−0.1018∗∗ (0.0274)	0.1901∗∗∗ (0.0282)	0.3318∗∗∗ (0.0334)	−0.0460 (0.0333)	0.3445∗∗∗ (0.0350)	0.2049∗∗∗ (0.0390)
*exercise*	0.0317∗∗∗ (0.0082)	0.0532∗∗∗ (0.0087)	0.0397∗∗∗ (0.0106)	0.0602∗∗∗ (0.0108)	0.0687∗∗∗ (0.0112)	0.0912∗∗∗ (0.0123)
*male*	0.0933∗∗∗ (0.0241)	0.1457∗∗∗ (0.0251)	0.0899∗∗ (0.0296)	0.0973∗∗ (0.0305)	0.1197∗∗∗ (0.0313)	0.1275∗∗∗ (0.0345)

∗ p < 0.05.

∗∗ p < 0.01.

∗∗∗ p < 0.001).

### 4.6 robustness test

Our regression analysis above illustrates the correlation pattern consistent with the argument that academic pressure lowers their sports activities. The survey data also provide information about students’ sports consumption and propensity to watch sports events. Nevertheless, there are two main concerns with our regression analysis.

1 Correlation does not imply causation. While the evidence is consistent with the consensus that older students exercise less because private lessons crowd out leisure time, correlation does not imply causation since the data does not include details on how students spend their leisure time or how many private lessons they take. In theory, older students may experience a change in taste towards sports and exercise less as they age. Moreover, parental controls loosen as students become and may exercise and train less without their parental pressure. In other words, there is more than one reason to explain why students exercise less over time.
2 Endogeneity concerns in years of schooling. The other main concern is that the *year of schooling* may correlate with other unobserved variables that affect exercise behavior, and sports consumption also correlates with years of schooling. However, *years of* schooling is not a choice for elementary and middle school students since the education policy requires at least nine years of schooling. So, while students can choose between the different types of schools (private vs. public), we assume that most students have to undergo some form of schooling even when they have no desire to take the entrance examination.
3 Outliers influencing the results*. Due to the substantial skewness in the sports-related expenditure*, there are concerns on whether extremely wealthy individuals might be driving the results behind this study. Since wealthy families may behave differently from the general population.

Regarding the first main concern, without data on the number of private lessons students take, we cannot rule out other explanations for why older students exercise, train less, and consume more. However, we can provide a partial test on the second concern because education is compulsory in the first nine years. Elementary and middle school students must go through a formal education system by law. The compliance rate is high since home-schooling is rare and child labor is illegal. Therefore for most students, education is not a choice for the first nine years. Consequently, we use only the dataset for elementary and middle school students and repeat the regression analysis of column 4 of Table 10, Table 11, Table 12, and column 5 of Table 13, presenting the results in Table 16 below. The result from Table 16 indicates a diminished impact of years of schooling on daily exercise, sports spectator, and sports expenditure. For years of schooling on daily exercise and sports expenditure, the effect is no longer statistically significant.

**Table 16:** Sports behavior at different years of schooling for primary and middle school students only. (Standard errors in parentheses.

Dependent Variable	*exercise*	*training*	*spectator*	*total expenditure*
*yos*	**−0.0042 (0.0055)**	**−0.0253**∗∗∗**(0.0017)**	**0.0056**∗∗∗**(0.0016)**	**0.0036 (0.0059)**
*training*	0.1820∗∗∗ (0.0279)	NA	0.3078∗∗∗ (0.0074)	0.8251∗∗∗ (0.0290)
*spectator*	0.2850∗∗∗ (0.0301)	0.3833∗∗∗ (0.0087)	NA	0.4240∗∗∗ (0.0314)
*exercise*	NA	0.0170∗∗∗ (0.0026)	0.0213∗∗∗ (0.0023)	0.0703∗∗∗ (0.0090)
*male*	0.1392∗∗∗ (0.0255)	0.0428∗∗∗ (0.0078)	0.0593∗∗∗ (0.0070)	0.0924∗∗∗ (0.0268)
*Pudong*	0.0565 (0.0355)	−0.0829∗∗∗ (0.0109)	−0.0728∗∗∗ (0.0099)	0.0068 (0.0373)
*suburban*	0.1274∗∗∗ (0.0329)	−0.1012∗∗∗ (0.0099)	−0.0670∗∗∗ (0.0092)	0.0295 (0.0341)
*remote-suburban*	−0.2052∗∗∗ (0.0373)	−0.0276∗ (0.0117)	−0.1600∗∗∗ (0.0096)	0.0926∗ (0.0402)

**p < 0.01.

∗ p < 0.05.

∗∗∗ p < 0.001).

**Fig. 2:**
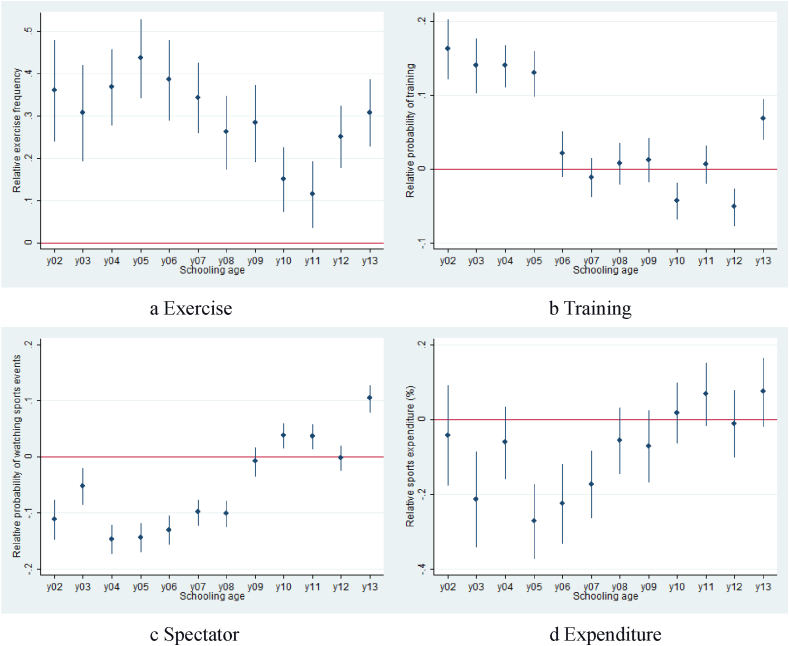
Examining the relative impact of year of schooling on the (a) frequency of exercise, (b) propensity to train, (c) propensity to watch sports events, and (d) the percentage of sports expenditure, normalizing grade 1 impact at 0 as the baseline.

At first glance, our main results seemed sensitive to the change in the dataset. However, to understand why the analysis results under compulsory education differ from our main results, we disaggregate the effects of years of schooling on frequency ofexercise behaviors. We conduct a regression by replacing the yos variable with twelve dummy variables from grades 2 to 13, with grade 1 as the benchmark. By doing so, we observe a trend in sports behavior and consumption at every schooling grade from elementary to the first year of college (grade 13). We plot the coefficients of all dummy variables in diagrams 2a to 2d, which represent the impact of years of schooling on sports behavior and consumption relative to grade 1. We use Fig. 2a as an example, highlighting year of schooling's effect on the number of daily exercise. We observe a fluctuating trend in which exercise level is high from grades 2 to 5, followed by a decline in the sixth grade. Students in grades 2 to 9 represent a mix of those who want to enter high school and those who do not. Those planning to enter high school face higher academic pressure than those not. Having both types of students diminishes the effect of academic pressure on sports behavior. By the tenth grade (first year of high school), the latter group drops out, leaving only those who face academic pressure. Therefore, although exercise is declining by the sixth grade, we notice a sharper drop in exercise levels in the tenth and eleventh grades, a discontinuity in the middle and high school cohorts. This discontinuity is less pronounced for the other variable of interest. For example, sports training declined earlier in grade 6 and remained low until after high school graduation, while the propensity to watch sports and spend on sports products increased since the sixth grade. Moreover, we observe the trend reverses with increased exercise and training activities upon finishing high school (grade 13), showing that students increased their exercise and training activities without academic pressure.

Our robustness test highlights the difference in behavior between two student cohorts under compulsory schooling, which diminishes the impact of academic pressure on reducing exercise and training activities. Moreover, the robustness test also shows that once high-school education becomes a choice, high school students show a significant drop in exercise and training, followed by a trend reversal in grades 13 when a student finishes high school. This trend proves that academic pressure cuts down on leisure time and lowers students' incentive to exercise and train.

Regarding the third concern for outliers driving out the underlying results, since sports-related expenditures are highly skewed towards high-income individuals, they may be driving the underlying results. We perform the same regressions after omitting individuals with extremely high incomes to show that the extreme outliers are not affecting our results. However, since we do not have data about high-income individuals, the alternative option is to drop household that spends more than 20,000 yuan per month on sports-related expenditures, which is an extreme amount considering that the average income in Shanghai is only 10,000 yuan per month. After removing the observation, Table 17 displays the result of spending more than 20,000 yuan monthly on sports-related expenditures. Table 18 replicates the results by dropping out families spending more than 10,000 yuan monthly on sports-related expenditures. Line 1 of Table 17, Table 18 shows that our main result still holds that exercise and training decrease with years of schooling while spectating sports events and total expenditure increase. By removing these outliers, we also observe two notable findings. First, by omitting high-expenditure households, the standard errors for most coefficients have been reduced, an unsurprising observation given that high-income families behave differently than the general household. Two, the coefficient for most district-related dummy variables (Pudong, suburban, and remote-suburban) is smaller. When we include high-income individuals, controlling for districts partially controls for family income since more affluent households usually prefer living in the urban districts; therefore, the district captures the difference in sports behavior between families of different incomes. Once we remove high-income households from our dataset, the difference in sports behavior between districts becomes smaller.

**Table 17:** Sports behavior at different years of schooling for expenditure below 20,000 yuan. (Standard errors in parentheses.

Dependent Variable	*exercise*	*training*	*spectator*	*total expenditure*
*yos*	**−0.0250**∗∗∗**(0.0026)**	**−0.0208**∗∗∗**(0.0008)**	**0.0181**∗∗∗**(0.0008)**	**0.0144**∗∗∗**(0.0025)**
*training*	0.2296∗∗∗ (0.0204)	NA	0.3725∗∗∗ (0.0056)	0.6086∗∗∗ (0.0192)
*spectator*	0.3585∗∗∗ (0.0212)	0.4190∗∗∗ (0.0061)	NA	0.3356∗∗∗ (0.0201)
*exercise*	NA	0.0208∗∗∗ (0.0018)	0.0289∗∗∗ (0.0017)	0.0591∗∗∗ (0.0059)
*male*	0.1767∗∗∗ (0.0180)	0.0163∗∗ (0.0054)	0.0162∗∗∗ (0.0051)	0.0781∗∗∗ (0.0174)
*Pudong*	0.0105 (0.0248)	−0.0812∗∗∗ (0.0074)	−0.0464∗∗∗ (0.0071)	0.0252 (0.0237)
*suburban*	0.0739∗∗∗ (0.0224)	−0.0706∗∗∗ (0.0068)	−0.0574∗∗∗ (0.0065)	0.0487∗ (0.0218)
*remote-suburban*	−0.1753∗∗∗ (0.0278)	−0.0246∗ (0.0086)	−0.1217∗∗∗ (0.0076)	0.0942∗∗∗ (0.0270)

∗ p < 0.05.

∗∗ p < 0.01.

∗∗∗ p < p0.001).

**Table 18:** Sports behavior at different years of schooling for expenditure below 10,000 yuan. (Standard errors in parentheses.

Dependent Variable	*exercise*	*training*	*spectator*	*total expenditure*
*yos*	−0.0257∗∗∗ (0.0027)	−0.0207∗∗∗ (0.0008)	0.0167∗∗∗ (0.0008)	0.0129∗∗∗ (0.0025)
*training*	0.2251∗∗∗ (0.0213)	NA	0.3595∗∗∗ (0.0058)	0.4938∗∗∗ (0.0190)
*spectator*	0.3539∗∗∗ (0.0222)	0.4095∗∗∗ (0.0065)	NA	0.2451∗∗∗ (0.0200)
*exercise*	NA	0.0201∗∗∗ (0.0019)	0.0277∗∗∗ (0.0017)	0.0502∗∗∗ (0.0058)
*male*	0.1748∗∗∗ (0.0186)	0.0134* (0.0056)	0.0576∗∗∗ (0.0052)	0.0546∗∗ (0.0171)
*Pudong*	0.0167 (0.0256)	−0.0771∗∗∗ (0.0076)	−0.0467∗∗∗ (0.0072)	0.0672∗∗ (0.0233)
*suburban*	0.0658∗∗ (0.0233)	−0.0687∗∗∗ (0.0070)	−0.0561∗∗∗ (0.0066)	0.0711∗∗∗ (0.0215)
*remote-suburban*	−0.1818∗∗∗ (0.0287)	−0.0219∗ (0.0088)	−0.1180∗∗∗ (0.0077)	0.01150∗∗∗ (0.0266)

∗ p < 0.05.

∗∗ p < 0.01.

∗∗∗ p < 0.001).

### 4.7 Paper's weakness

Our paper faces two major limitations.

1. **Data limitation:** Ideally, when measuring an individual's propensity to exercise at different levels of schooling, we would like to control for individual-specific interest in sports and their household income level because these variables are crucial in determining their level of exercise. However, the survey data doesn't have specific questions that tease out their income level and interest in exercise and sports. Therefore, we have to rely on proxy variables such as exercise frequency, expenditure in sports training, and watching sports activities to proxy for an individual preference for sports, and district level to proxy for individual household income. The usefulness of these proxy variables depends on how well they capture our variable of interest, and often, the effectiveness of these proxies is based on our assumption.
2. **Difficulty teasing out the causality:** While our paper shows that students exercise less when they approach closer to the entrance examination date, even with our robustness test in section 4.6, we could only show the correlation between these two variables. As mentioned in section 2.2, several factors explain the lower exercise and sports participation among Chinese adolescents. The most likely contender to our hypothesis is higher gaming and social media consumption. With cheaper mobile devices, more engaging video games, and social media app algorithms tailoring to individual viewing content, students spend significantly more time on their mobile devices and less on other leisurely activities, including exercise and sports. To be consistent with the existing data, we need to show that adolescents are more engaged in social media apps and online games as they age, which is plausible for older adolescents since parents typically give them greater autonomy in deciding on their leisure time. In other words, the correlation between academic pressure and less exercise could also be explained by the recent rise in adolescent's engagement in social media and online gaming. This issue has been an ongoing concern for the Chinese government since 2024 when it proposed to regulate the amount of time adolescents are allowed to spend on online games [35].

## 5 policy implication, potential issues, and conclusion

### 5.1 summary

Our findings indicate that.

1. Older students exercise less and participate less in paid sports training, consistent with the narrative that students have less leisure time from after-school programs to increase their chances of passing the high school and college entrance exams.
2. Older students watch more sports events. While watching sports events is time-intensive, mobile devices make it more flexible and allow students to watch sports highlights while engaging in other activities. Watching sports events has become a substitute for sports activities.
3. Older students spend more only on specific sports categories such as sports equipment, sports subscriptions, and tickets to sports events. While our finding is consistent with older students spending more time and money on sports events, we should be cautious with such an interpretation since the coefficients are minor and do not hold for all sports-related expenditures, with attire and footwear being the significant component of sports-related expenditure.

### 5.2 Policy implications and conclusion

The most important policy implication from our finding is that academic pressure reduces leisure time, which leads to lower sports activities. While academic pressure naturally exists in any education system, a more holistic education system that moves away from rote memory and standardized testing and promotes more practical skills and critical thinking may reduce the pressure on students who are not proficient with only one method of preparing for the test. With fewer standardized tests, families are less likely to rely on after-school programs to prepare for the exam.

Even though reducing academic pressure may increase exercise and sports participation, this may not work out in practice. By reducing academic pressure and freeing up adolescent leisure time, students naturally gravitate to the most enjoyable leisure activities, including using social media apps, browsing the Internet, and gaming. Therefore, public schools should promote the health benefits of exercise and provide straightforward guidelines for maintaining a healthy lifestyle. By promoting the benefits of exercise, families engage in sports activities on their initiative.

In recent years, the government has addressed the concerns of academic pressure by directly regulating the after-school program. In July 2021, the Chinese government introduced the "Double Reduction Policy" to "reduce homework and enrollment in after-school programs that directly tutor pressure on primary and secondary school students, reduce families' spending on expensive tutoring, and improve compulsory education" [27]. The policy intends to reduce the cost of education and stave off the decline in China's demography because education is an essential component of a child's upbringing. While it is too early to tell if the program achieved its intended goal, we observe an uptick in adolescent enrollment in extracurricular and sports-related activities since the "Double Reduction Policy" did not specifically restrict these programs.

The reception from the "Double Reduction Program" is mixed. On the one hand, some parents applaud the measure. They face less pressure to send their child to after-school programs, and it allows their kid more time for exercise and sports-related activities. On the other hand, private education companies experienced sharp adjustments to become non-profit entities (a government requirement) and reorient their lesson towards more extracurricular activities. While we agree with the government's position to regulate after-school programs, it fails to address the primary consumer's concern for insufficient education resources, which is the key drive behind the growth of after-school programs. Moreover, restricting after-school programs may increase the inequality between families with abundant educational opportunities and those without (low-income families). The government should promote health benefits and the importance of sports and exercise and provide parents with the necessary information to make an informed choice.

We conclude by outlining two areas for future research. First, the effect of academic pressure on students' propensity to undertake physical activities remains under-represented in the current literature, especially when various competing factors, such as a rise in social media and video games, correlate with a decline in sports participation [36]. Second, with the recent implementation of China's “Double Reduction Policy,” researchers should focus on assessing the long-term impact of this policy, from individual students' academic performance and their propensity to undertake physical activity to broader socioeconomic implications, e.g., does the “Double Reduction Policy” promote better social mobility? These two areas for future research will help us better understand individual students' exercise behavior and the effectiveness of more holistic educational policies.

## Data availability statement

The data associated with this study has not been deposited into a publicly available repository because the authors do not have permission from the Shanghai Sports Bureau, under the jurisdiction of the Shanghai Municipal Authority, to make this data publicly available.

## CRediT authorship contribution statement

**Hon Foong Cheah:** Writing.

## Declaration of competing interest

The authors declare that they have no known competing financial interests or personal relationships that could have appeared to influence the work reported in this paper.

## Footnotes

1 While the data used to construct the “300 Index” is not publicly available, reports from the “300 Index” are publicly available on these websites: https://www.sport.gov.cn/n20001280/n20745751/n20767239/c21692342/content.html, https://www.sport.gov.cn/n20001280/n20745751/n20767277/c21338791/content.html, https://cn.csgf.org.cn/xhzx/hydt//3827.html.

2 The former district of Zhabei is now administered under the inner district of Jingan [37].

3 We retrieve the map for Shanghai from the following website: https://fr.shanghaimap360.com/plan-quartier-shanghai.

4 The Tobit framework has been used by studies such as Ruseski et al. [37], Dawson and Downward (2013), and Thibaut et al. [38].

## References

[1] Kan Qian (30 August 2019). https://www.open.edu/openlearn/education/brief-introduction-the-chinese-education-system.

[2] Liu Y. (2016).

[3] Chen P., Wang D., Shen H., Yu L., Gao Q., Mao L., Jiang F., Luo Y., Xie M., Zhang Y., Feng L. (2020). Physical activity and health in Chinese children and adolescents: expert consensus statement (2020). Br. J. Sports Med..

[4] Aarts H., Paulussen T., Schaalma H. (1997). Physical exercise habit: on the conceptualization and formation of habitual health behaviours. Health Educ. Res..

[5] Downward P., Rasciute S. (2015). Exploring the covariates of sport participation for health: an analysis of males and females in England. J. Sports Sci..

[6] Eberth B., Smith M. (2010). Modelling the participation decision and duration of sporting activity in Scotland. Econ. Modell..

[7] Ruseski J.E., Maresova K. (2014). Economic freedom, sport policy, and individual participation in physical activity: an international comparison. Contemp. Econ. Pol..

[8] Bauman A., Bull F., Chey T., Craig C., Ainsworth B. (2009). The international prevalence study on physical activity: results from 20 countries. Int. J. Behav. Nutr. Phys. Activ..

[9] García J., Lera-López F., Suárez M.J. (2011). Estimation of a structural model of the determinants of the time spent on physical activity and sport: evidence for Spain. J. Sports Econ..

[10] Petilli M.A., Rinaldi L., Trisolini D.C., Girelli L., Vecchio L.P., Daini R. (2020). How difficult is it for adolescents to maintain attention? The differential effects of video games and sports. Q. J. Exp. Psychol..

[11] Kokolakakis T., Castellanos-García P., Lera-López F. (2017). Differences in formal and informal sports participation at regional level in England. International Journal of Sport Policy and Politics.

[12] Løyland K., Ringstad V. (2009). On the price and income sensitivity of the demand for sports: has Linder's disease become more serious?. J. Sports Econ..

[13] Downward P., Rasciute S. (2016). ‘No man is an island entire of itself’: the hidden effect of peers on physical activity: tohn Donne, Meditation XVII. Soc. Sci. Med..

[14] Adachi P.J., Willoughby T. (2016). Does playing sports video games predict increased involvement in real-life sports over several years among older adolescents and emerging adults?. J. Youth Adolesc..

[15] Downward P., Rasciute S. (2010). The relative demands for sports and leisure in England. Eur. Sport Manag. Q..

[16] Humphreys B.R., Ruseski J.E. (2011). An economic analysis of participation and time spent in physical activity. B E J. Econ. Anal. Pol..

[17] Downward P., Hallmann K., Pawlowski T. (2014). Assessing parental impact on the sports participation of children: a socio-economic analysis of the UK. Eur. J. Sport Sci..

[18] Xiang Zubing, Zhang Yanni, Luo Yunke, Shaocong &Zhao (2021). Effect of extracurricular physical exercise on teenagers' academic achievement—— an empirical research based on threshold regression model. J. Phys. Educ..

[19] Fang C., Huang B. (2021). Can physical exercise promote the development of teenagers' cognitive ability? An empirical study based on CEPS. J. East China Normal Univ. Educ. Sci..

[20] Liu J., He X., Zhang Y. (2021). Physical exercise, parent-teenager relationship and Teenager's psychological health——evidence from China education banel survey. China Youth Study.

[21] Lu C., Stolk R.P., Sauer P.J., Sijtsma A., Wiersma R., Huang G., Corpeleijn E. (2017). Factors of physical activity among Chinese children and adolescents: a systematic review. Int. J. Behav. Nutr. Phys. Activ..

[22] Liu M., Wang M., Xu J., Zhang Y. (2018). Analysis on the public policy of youth sports in China at the background of health China. China Sport Science.

[23] Yang Shoujian (2020). Research on teenagers' participation in sports. China Youth Study.

[24] Yu Chengxi (2021). Discussion on the development direction and path of youth sports in the Context of healthy China. Journal of Guangzhou Sport University.

[25] Dong Baolin, Lijuan &Mao (2021). The relationship between school natural environment, interpersonal environment and adolescents' physical exercise. J. Phys. Educ..

[26] Xue H., Ding X. (2009). A study on additional instruction for students in cities and Towns in China. Educ. Res..

[27] Xue E., Li J. (2023). What is the value essence of “double reduction”(Shuang Jian) policy in China? A policy narrative perspective. Educ. Philos. Theor..

[28] Li Y. (2024, February 22). Average cost of childrearing in China among world's highest: think tank report. The Global Times.

[29] Kristof N.D. (2011, January 15). China's winning schools?. The New York Times.

[30] Kirkpatrick R., Zang Y. (2011). The negative influences of exam-oriented education on Chinese high school students: Backwash from classroom to child. Lang. Test. Asia.

[31] Fu, Y. China's Unfair College Admissions System. Atlantic. Retrieved from https://www.theatlantic.com/china/archive/2013/06/chinas-unfair-college-admissions-system/276995/.

[32] Becker G.S. (1964).

[33] Wooldridge J.M. (2010).

[34] Tobin James (1958). Estimation of relationships for limited dependent variables. Econometrica: J. Econom. Soc..

[35] Ye J. (2023, December 22). https://www.reuters.com/world/china/china-issues-draft-rules-online-game-management-2023-12-22/.

[36] Henchoz Y., Studer J., Deline S., N'Goran A.A., Baggio S., Gmel G. (2016). Video gaming disorder and sport and exercise in emerging adulthood: a longitudinal study. Behav. Med..

[37] Ruseski J.E., Humphreys B.R., Hallmann K., Breuer C. (2011). Family structure, time constraints, and sport participation. European Review of Aging and Physical Activity.

[38] Thibaut E., Eakins J., Vos S., Scheerder J. (2017). Time and money expenditure in sports participation: the role of income in consuming the most practiced sports activities in Flanders. Sport Manag. Rev..

